# Blunt force homicides in Denmark 1992–2016

**DOI:** 10.1111/1556-4029.15118

**Published:** 2022-08-18

**Authors:** Asser H. Thomsen, Peter M. Leth, Hans P. Hougen, Palle Villesen

**Affiliations:** ^1^ Department of Forensic Medicine Aarhus University Aarhus N Denmark; ^2^ Department of Forensic Medicine University of Southern Denmark Odense C Denmark; ^3^ Department of Forensic Medicine, Faculty of Health Sciences University of Copenhagen Copenhagen Denmark; ^4^ Bioinformatics Research Centre Aarhus University Aarhus C Denmark; ^5^ Department of Clinical Medicine Aarhus University Aarhus Denmark

**Keywords:** assault, autopsy, blunt force trauma, Denmark, forensic pathology, head injury, homicide, interpersonal violence

## Abstract

Blunt force trauma is a common homicide method, inflicted in three different ways: bodily force, assault with blunt objects of various types and falls from height. The objective of this study is to provide thorough information on blunt force homicides with data on the victims, the offenders, the surrounding circumstances, the injury methods, the extent of injuries, and survival time, which will help inform the inexperienced as well as the seasoned forensic pathologist in their daily work with death investigation and as expert witnesses in court. We have analyzed autopsy reports and available case files of 311 blunt force homicides, making up 21.9% of all homicides in Denmark during 1992–2016. Most victims and offenders were male. Altercation in the setting of nightlife and intoxication was common in male victims, while most female victims were killed in a domestic setting. Bodily force was the most common primary homicide method, followed by assault with a blunt object and fall from height. The head was the region that most often had external injuries, with no noteworthy difference between cases with bodily force and blunt objects. Two out of three victims had one or more lacerations, most often located on the head and more often on the front. Brain injury was the primary cause of death in at least 72.0% victims. Compared to bodily force victims of blunt object assault were especially prone to skull and brain injuries, had a higher trauma score, and more died at the crime scene and had a shorter survival time.

**Highlights**

- Blunt force injury caused 22% of homicides (311 deaths) in Denmark during 1992–2016.
- Bodily force was most commonly used in blunt force homicide, followed by assault with blunt object.
- Brain injury was the primary cause of death in three out of four blunt force homicides.
- Blunt object assault had higher trauma scores and shorter survival time than bodily force assault.
- Male victims of blunt force homicide were often killed in the setting of nightlife and intoxication.
- Most female victims of blunt force homicide were killed in a domestic setting by an intimate partner.

## 1 INTRODUCTION

Blunt force trauma is often used in interpersonal violence and is a common homicide method [1, 2, 3, 4, 5, 6, 7, 8, 9, 10, 11, 12, 13]. In blunt force homicide, the blunt force trauma is inflicted in three different ways:

1. Bodily force in form of punches, kicks, stomping, throws, etc.
2. Assault with blunt objects of various types, and
3. Falls from height.

Blunt force trauma is used in other homicide methods, such as traumatic asphyxia and assault with an automobile, but those types are usually not included in studies of blunt force homicides [4].

Blunt force trauma causes external injuries in form of abrasions, contusions, and lacerations of the skin and internal injuries such as fractures, intracranial hemorrhages, and damage to internal organs [1, 2]. The injury pattern and injury severity in a victim can help determine the method of injury and the manner of death. In assaults by bodily force and with blunt objects, part of the method of injury can be an accompanying fall, and in the case of an unyielding surface, often with significant brain injury [1, 2]. As these passive blunt force injuries result from an active blunt force trauma, such deaths are – from a medical point of view – considered as homicides.

Even though blunt force trauma is often encountered in forensic pathology, and is an easily accessible homicide method, there are surprisingly few scientific studies on the subject, as noted by Verzeletti et al [12]. To fill this gap, we will examine the findings in all victims of blunt force homicide in a 25‐year period in Denmark, with a special focus on injuries in victims of bodily force assault and blunt object assault. The objective of the study is to provide thorough information on blunt force homicides with data on the victims, the offenders, the surrounding circumstances, the injury methods, the extent of injuries, and survival time, which will help inform the inexperienced as well as the seasoned forensic pathologist in their daily work with death investigation and as expert witnesses in court.

## 2 MATERIALS AND METHODS

From the databases of the three departments of forensic medicine in Denmark, we identified all homicides in the period 1992–2016 [3]. The average population size in Denmark was 5.41 (5.16–5.71) million people [3]. Homicides with blunt force trauma as the primary homicide method were selected for further analysis.

For each homicide, we registered general information about the homicide, the victim, and the offenders in one database, including weapon type and survival time. The homicides were grouped based on the typology of the European Homicide Monitor, which emphasizes the importance of victim‐offender relation [14]. In a second database, we registered every injury on the skin and in organs using the Abbreviated Injury Scale (AIS) framework with localizers [15, 16, 17]. One author (AHT) has completed the AIS‐training program and collected all the data. From the AIS‐data, we retrieved the number of lacerations in each victim and the Injury Severity Score (ISS). The normal grouping of ISS is “minor (1–3),” “moderate (4–8),” “serious (9–15),” “severe (16–24),” and “critical (25–75)” [18]. To better suit the homicide victim population that has few victims with low scores, we define three ISS categories: “low (1–24),” “medium (25–44),” and “high (45–75)”.

We registered the data electronically in EpiData (EpiData Association, 2010, Odense, Denmark; http://www.epidata.dk) with double entry of the AIS‐data. We exported the data to Stata (StataCorp. 2015. Stata Statistical Software: Release 14.; StataCorp LLC.) and Rstudio (RStudio Team [2021]. RStudio: Integrated Development for R. RStudio, Inc.; http://www.rstudio.com/) for statistical analysis and data visualization.

We analyzed annual data with linear regression, using lm() in R. We fitted models allowing for different regression lines using lm(number ~ year). For differences between groups (e.g., difference in mean age between female and male victims), we used permutation tests of 100,000 permutations. For each permutation, we permuted the sex and calculated the mean difference between the two groups (the null). Contingency tables were tested with the chi‐squared test. For survival data, we used the Cox proportional hazards model, including adjustment for primary methods of blunt force trauma using the “survival” package in R.

The study has been approved by the Danish Data Authority.

## 3 RESULTS

### 3.1 Blunt force homicides

Of the 1417 homicides in Denmark from 1992–2016, 311 (21.9%) had blunt force trauma as the primary homicide method. Other homicide methods contributed to the death in 33 (10.6%) of those homicides, mostly asphyxia or sharp force trauma.

Blunt force trauma contributed to the death in 3.8% of the remaining 1106 homicides with a primary homicide method other than blunt force trauma, mostly asphyxia or sharp force trauma.

The blunt force trauma homicides were committed in 307 events, distributed in 302 events with one victim and 5 events with multiple victims (9 victims, 2–4 victims per event). There was an average of 12.4 (range: 5–21) blunt force homicides per year and they exhibited a significant reduction of 0.38 homicides per year (linear regression: *p* < 0.01, *F* = 17.0, *R* ^2^ = 0.43) (Figure S1).

**FIGURE 1:**
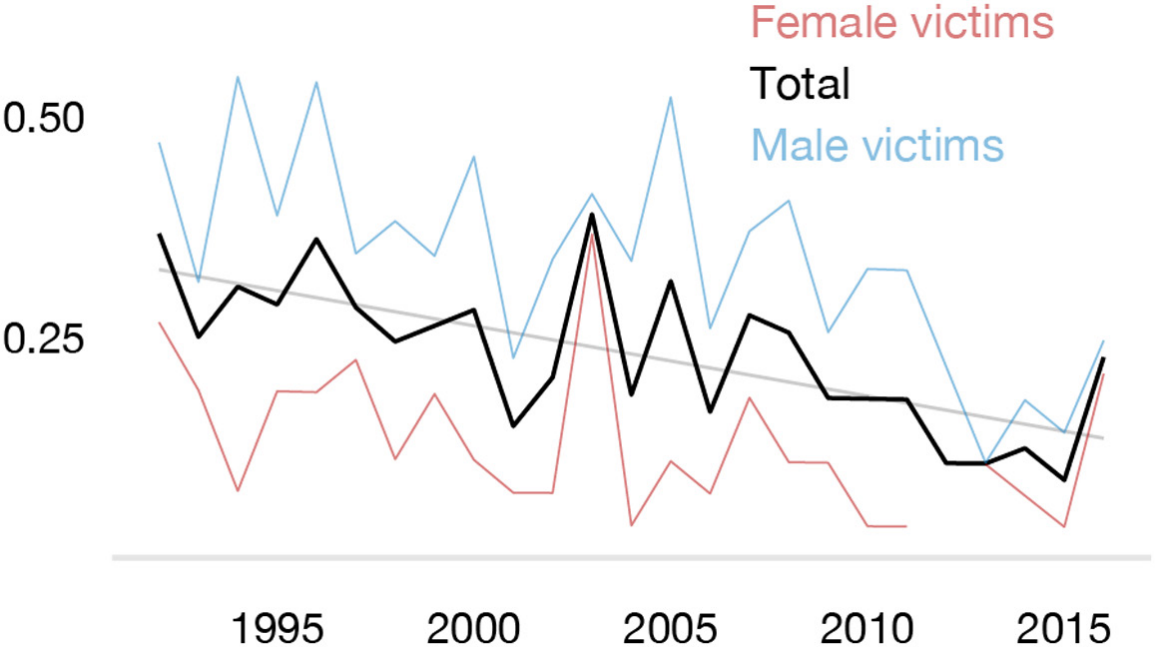
Blunt force homicide rate (per 100,000) 1992–2016.

The corresponding rate was 0.23 per 100,000 with a significant annual decrease of 0.008 per year (linear regression: *p* < 0.001, *F* = 21.7, *R* ^2^ = 0.49) (Figure 1). The rate was higher in men (0.34 per 100,000) than women (0.13 per 100,000) and both sexes showed a significant decrease (male victims: slope = −0.011, *p* < 0.001, *F* = 92.3, *R* ^2^ = 0.45) and female victims (female victims: slope = −0.005, *p* < 0.001, *F* = 26.6, *R* ^2^ = 0.19).

### 3.2 The victims and the offenders

**FIGURE 2:**
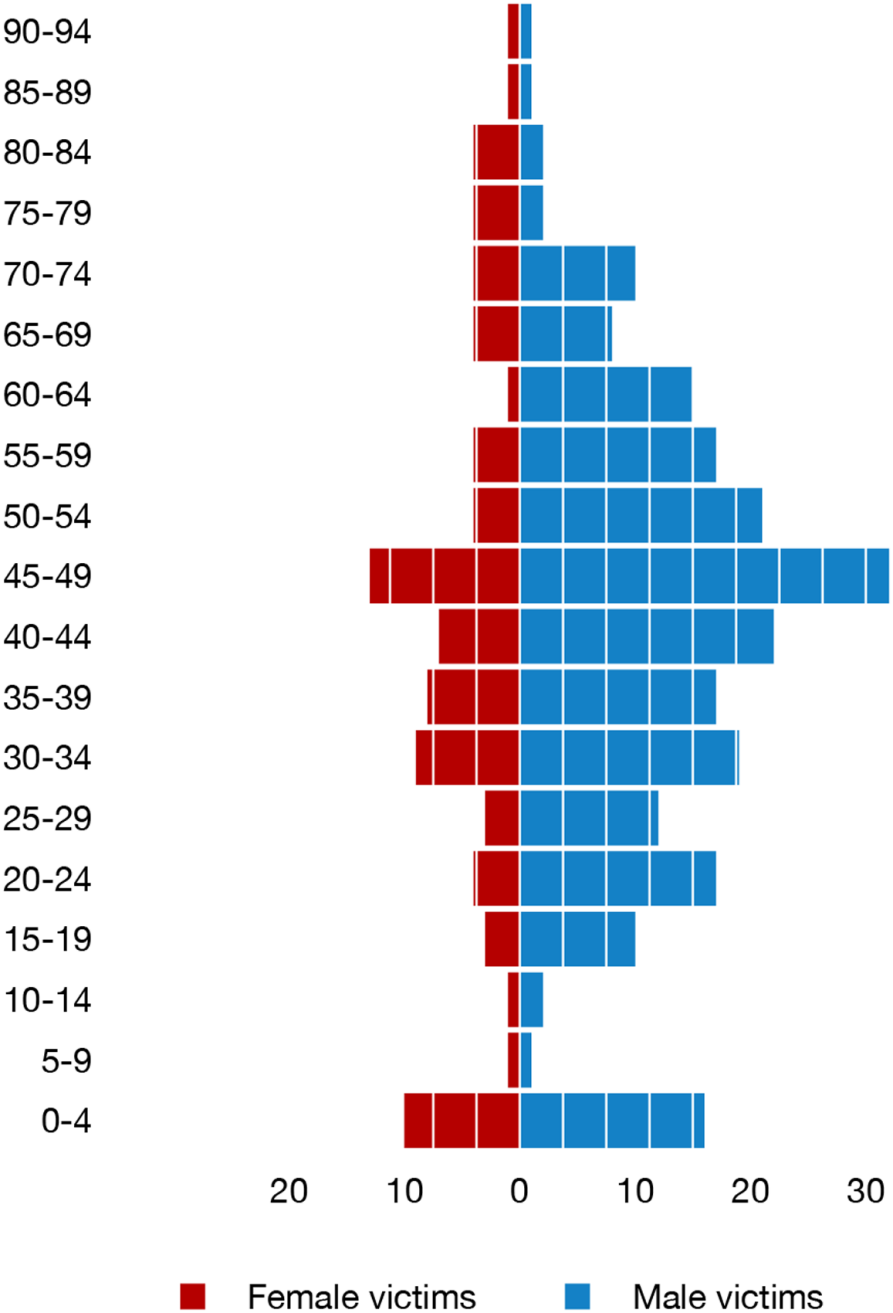
Age‐sex pyramid for blunt force homicide victims. The bars show the number of homicides for each age group in 5‐year intervals.

Most victims were male (225 (72.4%) males vs. 86 (27.6%) females). The mean age of the victims was 41.6 years (0–91, SD = 20.8, median = 43) (Figure 2), 41.5 years (0–90 SD = 19.5, median = 43) for males, 42.1 years (0–91, SD = 24.0, median = 42.5) for females, with no significant differences between sexes (Permutation test, *p* < 0.80). Children under the age of 1 year made up 17 (5.5%) of the victims (58.8% boys, 41.2% girls), while children under the age of 15 made up 31 (10.0%) of the victims (61.3% boys, 38.7% girls). All but two of the children under the age of 15 were killed by a parent/stepparent, the father being the sole offender in 16 homicides, the father with possible involvement of the mother in four homicides, and the mother as the sole offender in four homicides. The annual number of blunt force homicides in children under 15 years has varied between zero and four with no discernable trend.

The offender's sex was known in 289 (92.9%) homicides, with 266 (92.0%) of those having only male offenders. In 70 (24.2%) of the homicides with known offender sex there were multiple offenders, and none were female‐only (Figure S2).

There were 209 homicides (205 homicide events) with one offender, where the age and sex of the offender were known. The mean age of these offenders was 34.8 years (15–82, SD = 12.6, median = 34), 34.7 years (15–82, SD = 12.6, median = 33.5) for males, 36.4 years (15–58, SD = 12.7, median = 36) for females (Figure S3), with no significant difference between the sexes (Permutation test, *p* < 0.92).

### 3.3 Typology and injury method

**FIGURE 3:**
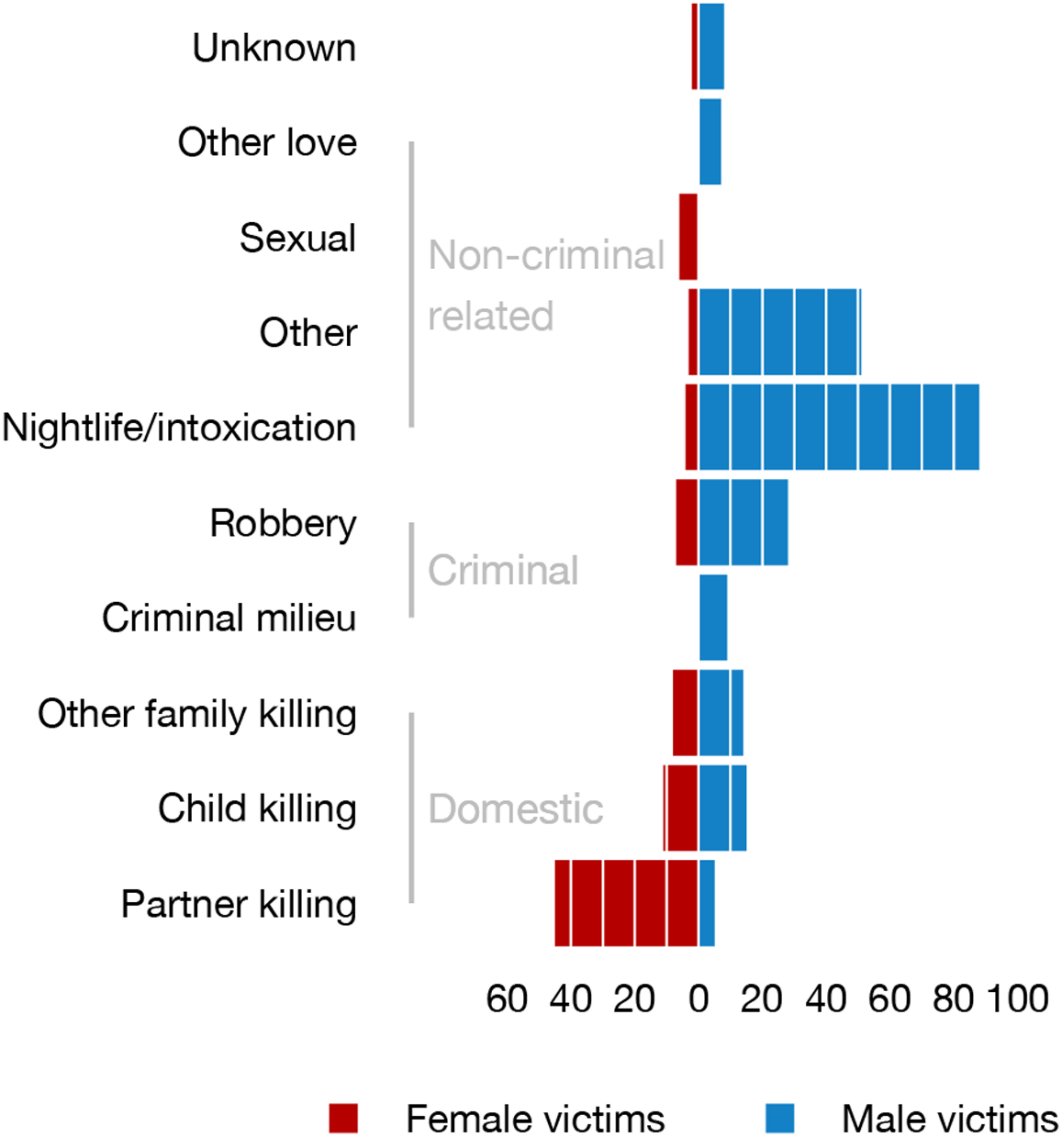
Homicide type related to sex of victim. The bars show the number of homicides for each main group.

There was a highly significant association between victim sex and homicide type (*χ* ^2^ = 162.06, df = 9, *p* < 0.001) (Figure 3, Table 2). Altercation in the setting of nightlife and intoxication was common in male victims (39.1% of male victims) often among friends/acquaintances. Most female victims (74.4% of female victims) were killed in a domestic setting, typically violence from a current or former intimate partner (52.3% of female victims) (Figure 3).

Two out of three homicides with bodily force occurred at an inside location vs. three out of four for homicides with blunt objects (Figure S4) (Table 1). Female victims were more commonly killed at an inside location (79.1% vs. 62.2%), reflecting the domestic setting of most homicides with female victims (Figure S5).

**TABLE 1:** Findings in victims of blunt force homicide, with homicide methods in columns

	Total (%)	Bodily force (%)	Blunt object (%)	Fall from height (%)
Number of victims	311	186 (59.8)	115 (37.0)	10 (3.2)
Inside location, %	70.0	67.1	79.5	10.0
Laceration, %	67.2	56.5	84.4	70.0
Number of lacerations, mean (min–max, median)	4.7 (0–50, 2)	2.0 (0–17, 1)	9.0 (0–50, 6)	4.4 (0–16, 2.5)
Defensive injuries, %	32.2	19.4	53.9	20.0
Cause of death, head, %	72.0	65.1	82.6	80.0
Skull fracture, %	65.0	50.5	87.0	80.0
Intracranial injury, %	74.3	68.3	83.5	80.0
EDH, %	3.5	3.2	4.3	0.0
SDH, %	35.7	46.8	19.1	20.0
SAH, %	34.4	32.8	37.4	30.0
Contusion, %	34.1	33.9	35.7	20.0
Laceration, %	20.3	5.4	41.7	50.0
Rib fractures, %	33.8	39.8	23.5	40.0
ISS – Low (1–24), %	61.4	65.1	58.3	30.0
ISS – Medium (25–44), %	33.8	32.8	36.5	20.0
ISS – High (45–75), %	4.8	2.1	5.2	50.0
Died at crime scene, %	55.0	41.4	76.5	60.0

Abbreviations: EDH, extradural hematoma; SDH, subdural hematoma; SAH, subarachnoid hemorrhage.

**TABLE 2:** Types of homicide, main groups, and subgroups

Type	Rate*	All *n* (%)	Males *n* (%)	Females *n* (%)
Domestic	0.07	98 (32%)	34 (15.1%)	64 (74.4%)
Partner killing	0.04	50 (16.10%)	5 (2.20%)	45 (52.3%)
Child killing	0.02	26 (8.40%)	15 (6.70%)	11 (12.8%)
Other family killing	0.02	22 (7.10%)	14 (6.20%)	8 (9.3%)
Criminal	0.03	44 (14%)	37 (16.4%)	7 (8.1%)
Criminal milieu	0.01	9 (2.90%)	9 (4.00%)	0 (0.0%)
Robbery	0.03	35 (11.30%)	28 (12.40%)	7 (8.1%)
Non‐criminal related	0.12	159 (51%)	146 (64.9%)	13 (15.1%)
Nightlife/intoxication	0.07	92 (29.60%)	88 (39.10%)	4 (4.7%)
Other	0.04	54 (17.40%)	51 (22.70%)	3 (3.5%)
Sexual	<0.01	6 (1.90%)	0 (0.00%)	6 (7.0%)
Other love	0.01	7 (2.30%)	7 (3.10%)	0 (0.0%)
Unknown	0.01	10 (3%)	8 (3.6%)	2 (2.3%)
Total	0.23	311 (100%)	225 (100%)	86 (100%)

* Per 100,000/year.

Bodily force was the most common primary homicide method (59.8%), followed by assault with a blunt object (37.0%) and fall from height (3.2%) (Figure S6, Table 1). As fall from height is a rare homicide method, the results will not be specified besides in Table 1 and in certain figures. About 10% of victims killed with bodily force as primary homicide method were also subjected to potentially lethal violence from blunt objects, and vice versa. Of the 301 victims that were killed from active blunt force trauma at least 10.0% had potentially lethal injuries sustained from a fall in conjunction with the trauma, most from standing height.

**FIGURE 4:**
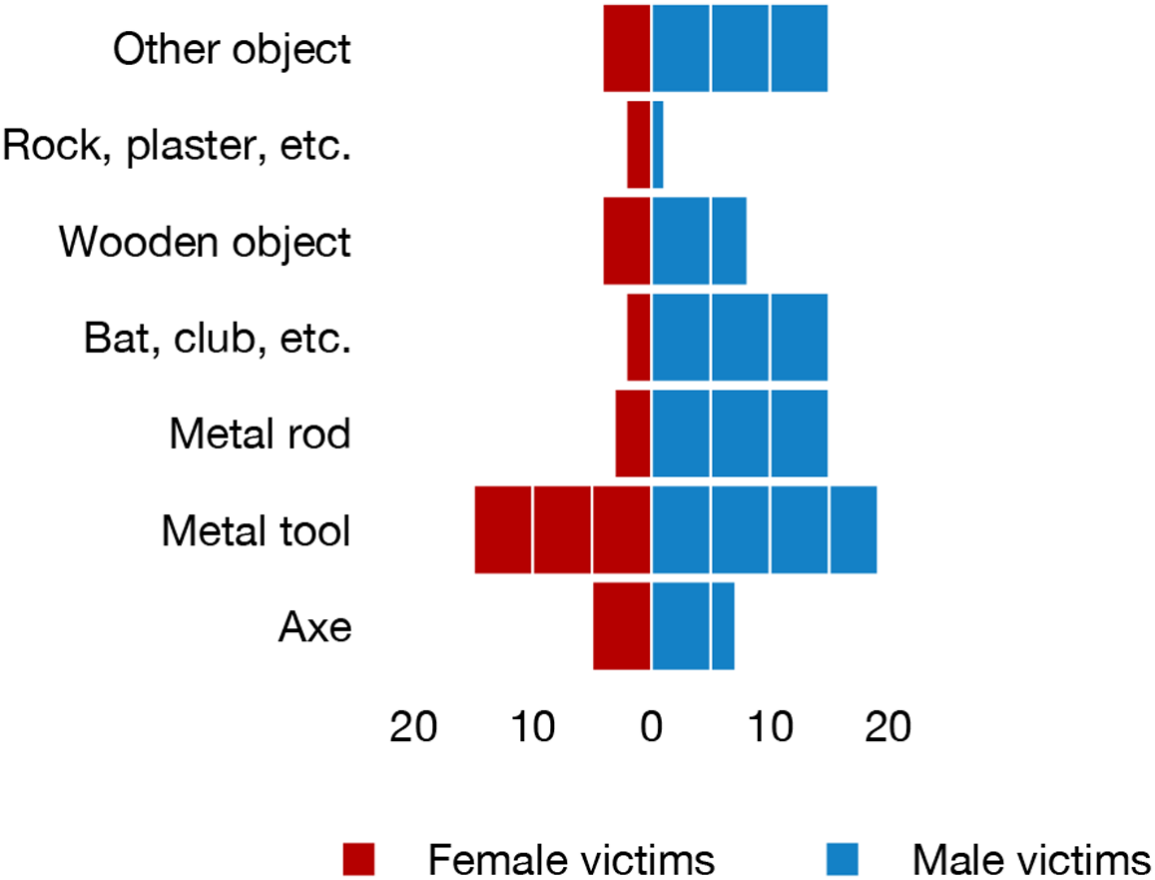
Type of weapon in blunt object homicides related to sex of victim. The bars show the number of homicides for each main group.

Various types of objects were used as weapons, with metal objects such as axes, other tools, rods, and bats being the most common (Figure 4).

### 3.4 Extent of injuries

**FIGURE 5:**
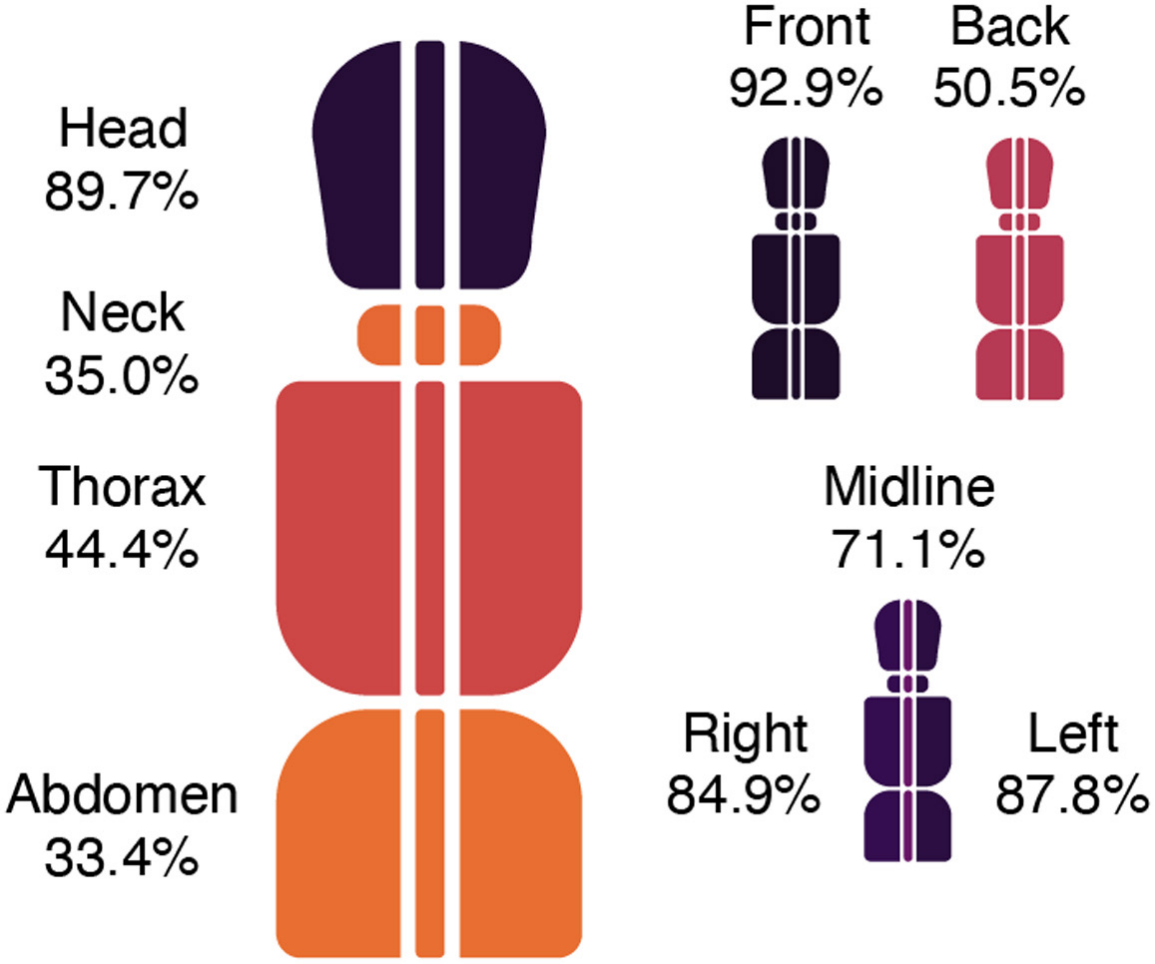
Distribution of contusions, abrasions, and lacerations relative to all 311 victims of blunt force homicide, i.e., the percentage of victims that have at least one lesion in a given area.

**FIGURE 6:**
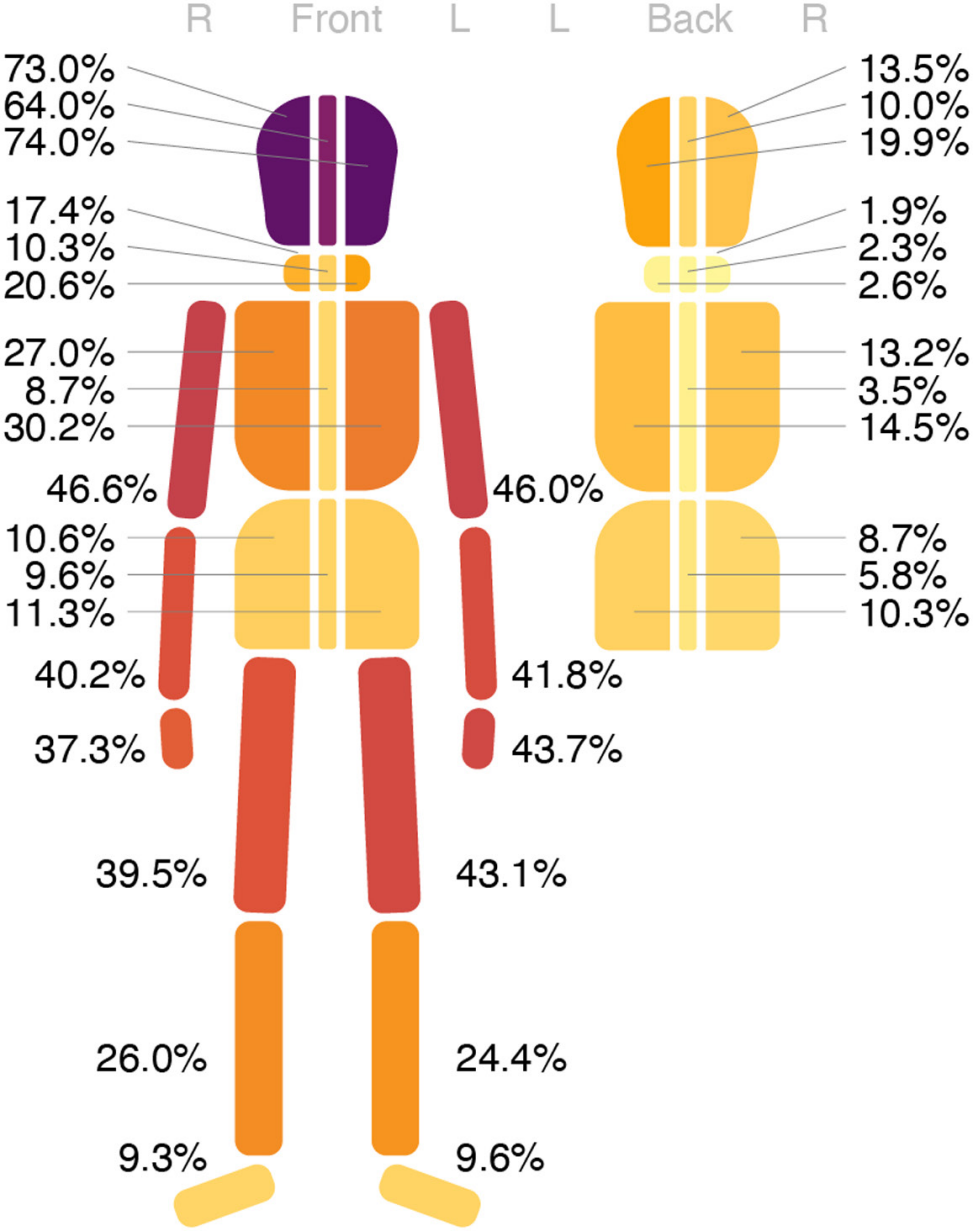
Distribution of contusions, abrasions, and lacerations relative to all 311 victims of blunt force homicide, i.e., the percentage of victims that have at least one lesion in a given area. Due to their mobility the extremities have not been separated into front and back.

External injuries from blunt force trauma in the form of contusions, abrasions, and lacerations were more common on the front of the body, than on the back (Figures 5 and 6). Not surprisingly, the head was the region that most commonly had external injuries (9 in 10 victims), with no noteworthy difference between cases with bodily force and blunt objects (Figure 6, Figures S7–S8). The neck, thorax, abdomen, and extremities more often had external injuries in cases with bodily force than blunt objects (Figures S9–S10).

Two out of three victims had one or more lacerations, most commonly and in higher numbers in deaths from blunt objects than bodily force (Table 1). Most lacerations were located on the head and more often on the front (Figure S11). Lacerations to the back part of the head and to the extremities were more common in victims of blunt object trauma than bodily force trauma (Figures S12–S13).

One‐third of the victims had what was interpreted as defensive injuries, i.e., from the victim trying to ward off attacks with arms and hands, and they were most commonly seen in deaths from a blunt object (Table 1, Figure S14). There were no differences regarding victim sex and prevalence of defensive injuries (31.1% of male victims vs. 34.9% of female victims [*χ* ^2^ = 0.16741, df = 1, *p* = 0.68]).

**FIGURE 7:**
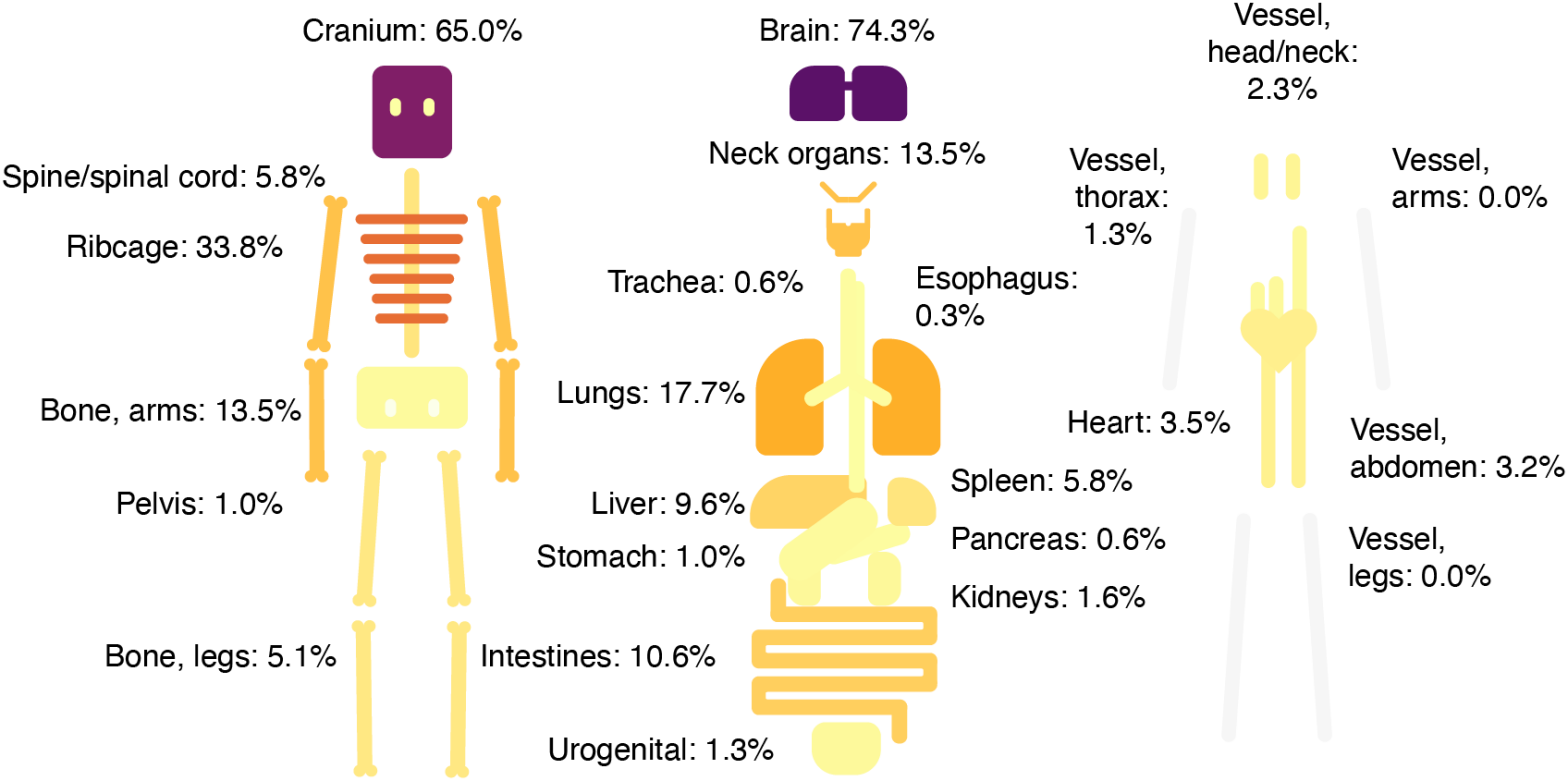
Distribution of injuries to organ systems, relative to all 311 blunt force homicides, i.e., the percentage of victims that have at least one injury in a given organ.

Brain injury was the primary cause of death in at least 72.0% victims. Skull fractures and injuries related to the brain were very common (Figure 7, Table 1). Victims of blunt object assault were especially prone to skull and brain injuries (Table 1, Figures S15–S16). Rib fractures (excluding from resuscitation efforts) were more common in bodily force assaults (Table 1). There was an aspiration of blood to the deep airways in 17.0% of victims and of gastric contents in 6.1%. The classical pure traumatic subarachnoid hemorrhage from rupture of a vertebral artery at the craniocervical junction with limited trauma was seen in 10 (3.2%) victims, all died from bodily force and were under the influence of alcohol.

**FIGURE 8:**
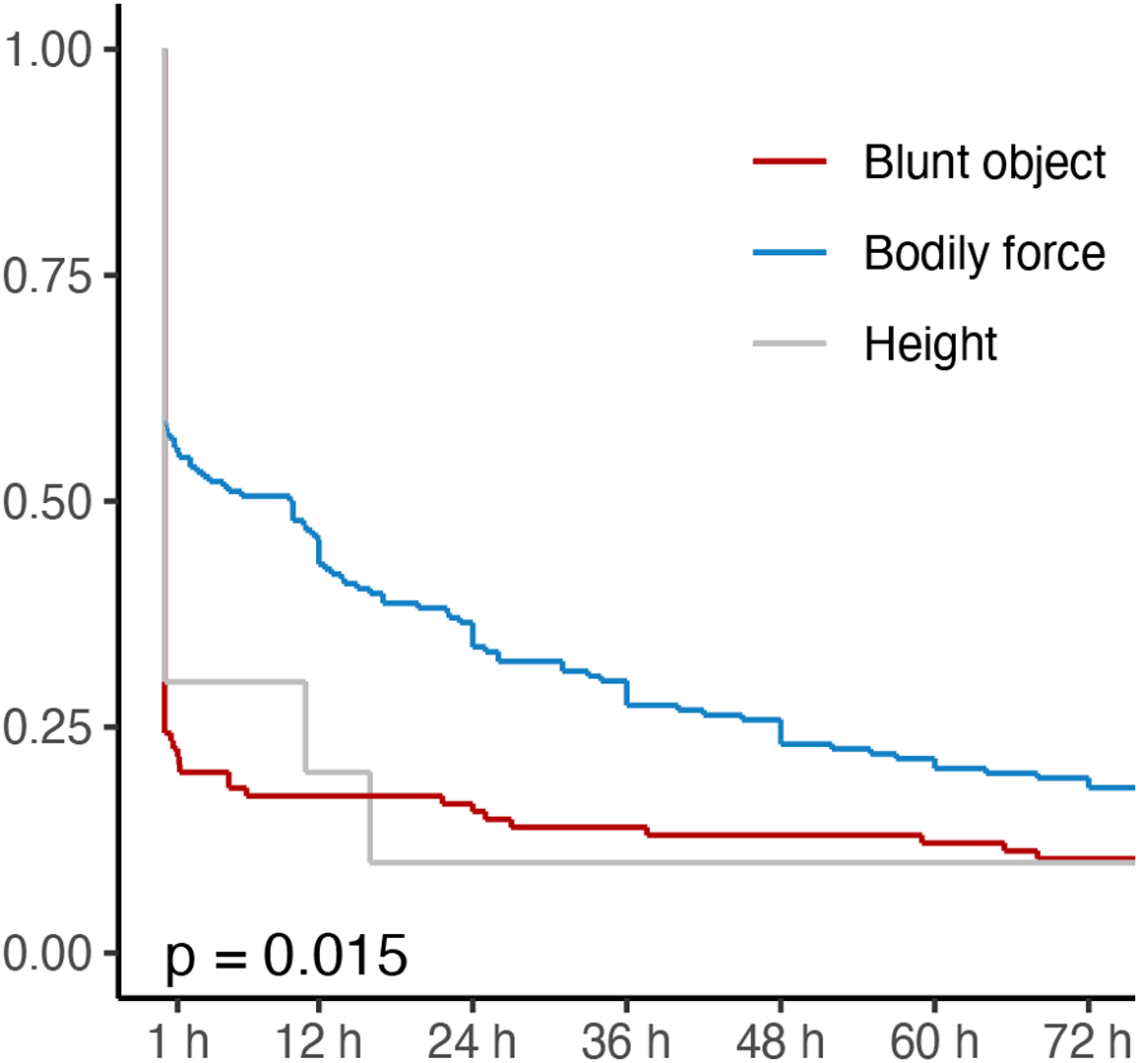
Survival curves for the first the 72 h, grouped by victims killed with bodily force, blunt objects, and fall from height.

Trauma scores and survival time depended on both the homicide method and the sex of the victims. Victims of blunt object assault had a higher trauma score (ISS) than bodily force, more died at the crime scene (Table 1, Figure S17) and the survival time was significantly shorter (*p* < 0.05) (Figure 8). Female victims had a higher trauma score than male victims (Figure S18) and the survival time was significantly shorter (Figure S19). Nine (2.9%) victims survived 1 month or more, while only one (0.3%) victim survived more than 1 year (died from complications from traumatic brain injury after 3 years).

## 4 DISCUSSION

Blunt force trauma is one of the four most common homicide methods in Denmark, and like sharp force trauma, gunshots and asphyxia, the homicide rate decreased in the studied 25‐year period as part of a general trend in homicides in the western world [3, 17, 19, 20, 21, 22]. The decreasing trend in homicide rates in seven northwestern European countries (including Denmark) during 1992–2016 affected all types of homicide, with the decline in female victimization being most prominent in domestic homicides, while the decline in male victimization was most prominent in non‐domestic homicides involving working‐aged men [22]. Possible explanations for the declining homicide rates are less violent crime in general, firearm restrictions, fewer weapons in public spaces, changes in lifestyle with more time spent at home, changes in alcohol culture and decreasing youth populations [3, 17, 19, 20, 21, 22]. Technological advances such as improved health care and telecommunication, has led to better and faster trauma treatment, improving survival rates in victims of violence [3, 17, 19, 20, 21, 22]. While the availability of knives and firearms in public can be controlled via legislation, this is not possible for all of blunt objects and certainly not for fists, knees, and feet, so the reduction in blunt force homicides must be part of a general trend in violence and perhaps also influenced by faster and better trauma treatment [16, 23].

On a global scale more than half of all homicides are committed with firearms [24, 25, 26]. In areas with high homicide rates most homicides involve firearms [24, 25, 26]. One example from the western world is South Carolina, USA, where the annual homicide rate for 2013–2018 was more than 8 per 100,000 and homicides by firearms represented 82% of all homicides [13]. In the same area, homicides by blunt force trauma represented 9% of all homicides, roughly translating into an annual blunt force homicide rate of 0.7 per 100,000, i.e., more than twice as high as found in our study (0.23 per 100,000) [13]. So, while blunt force homicides in certain areas make out a small proportion of all homicides, it still represents a significant number of deaths, compared to areas with lower homicide rates. In a study from Rome, Italy during 2000–2014 (with an annual homicide rate of around 0.5 per 100,000 and where homicides by firearms represented 39% of homicides), blunt force trauma represented 20% of homicides, roughly translating into an annual blunt force homicide rate equal to the rate in our study [27].

As with homicides by sharp force trauma and gunshots the majority of victims and most offenders were male [17, 19]. Males tend to be more impulsive, aggressive, and violent than females. Like other homicide methods we found a significant difference between the type of homicide in relation to victim sex and the homicide location [3, 17, 19, 20]. Most female victims were killed in a domestic setting, often by a current or former partner, and many male victims were killed by a friend/acquaintance during altercations in the setting of nightlife and intoxication. For both sexes, this is similar to other common homicide methods in Denmark [3, 17, 19, 20].

It is not surprising that bodily force is the most common method in blunt force homicides, as the body is always available as a weapon [1, 2]. In Denmark, bodily force injuries are much more common in survivors of blunt force trauma, than injuries from blunt objects [28, 29]. In homicides, however, blunt object injuries are relatively common. This reflects the blunt objects potential for inflicting serious injuries, which is confirmed by the ISS being higher, more victims dying at the crime scene, and survival time being shorter in victims dying from blunt object assault compared to victims dying from bodily force injuries.

That the head and the brain often are injured in blunt force homicides is well known from previous studies [4, 5, 30, 31, 32]. The brain is vulnerable to direct injury during the infliction of the primary blunt force trauma and from the related fall to the ground, as well as the secondary effects of swelling and hemorrhage in the closed cranial cavity [1, 2]. The injuries in the brain can lead to a lowered level of consciousness, where aspiration of blood and stomach contents can compound the effects on the victim, leading to death from injuries that in themselves are not considered directly lethal.

The homicide victims who died from blunt object trauma had fewer injuries to sites other than the head, compared to victims of bodily force. A possible explanation for this could be that an assailant using a blunt object as a weapon is more focused on inflicting head trauma, compared to an assailant using bodily force. Furthermore, the severe trauma from blunt object attacks will quicker lead to unconsciousness, preventing the victim from continuing movement and struggle.

We do not provide data on the number of abrasions and contusions, as it almost impossible to interpret whether multiple injuries in an area are from a single impact or multiple impacts. We do, however, provide such data for lacerations, as each laceration more often can be correlated to a single impact, and thus to the degree of inflicted violence, as seen from the offender's perspective. The higher number of lacerations and injuries interpreted as of a defensive nature in blunt object trauma compared to bodily force trauma must both be related to the more severe nature of the injuries and the unyielding nature of the blunt objects [1, 2]. That lacerations to the back of the head are more common in blunt object trauma could be explained simply from lacerations being more common in that type of trauma.

### 4.1 Limitations

In Denmark, all homicide victims are required by law to undergo medicolegal autopsy as part of the death investigation, which is why we have chosen the databases of the three departments of forensic medicine in Denmark for identifying relevant homicide cases [3]. Can we be certain that all blunt force homicides have been reported to the medicolegal system? In certain situations, it can be difficult to interpret the findings in a deceased person dying from blunt force trauma [1, 2]. A classical problem is whether injuries are the result of a primary fall or precipitated by an assault, and it would be naïve to think that all deadly assaults are recognized as such, especially with complicating factors, such as alcohol intoxication and putrefaction [1, 2]. Delayed deaths from assault also represent a challenge to the medicolegal system and can go unreported, as care providers can fail to recognize the link between death from an apparent disease process, such as pneumonia or a seizure disorder, and remote traumatic injury [33]. Most victims of blunt force homicide in our study died within a month, and only one victim survived more than a year, but we have no way of knowing how many true homicide victims dying remotely might have been missed. Although the remote deaths and the misinterpreted deaths from assault represent a limitation of our study, we believe that most homicides from blunt force trauma have been included in our study.

## 5 CONCLUSION

Blunt force trauma was a common homicide method in Denmark during 1992–2016. Two out of three victims were male, while more than 9 out of 10 offenders were male, with a highly significant association between victim sex and homicide type. Altercations in the setting of nightlife and intoxication was common in male victims, while most female victims were killed in a domestic setting, typically violence from a current or former intimate partner.

Bodily force was the most common primary homicide method, followed by assault with a blunt object and fall from height. The head was the region that most commonly had external injuries, with no noteworthy difference between cases with bodily force and blunt objects. Two out of three victims had one or more lacerations, most commonly and in higher numbers in deaths from blunt objects than bodily force. Most lacerations were located on the head and more often on the front. Brain injury was the primary cause of death in at least 72.0% victims. Compared to bodily force, victims of blunt object assault were especially prone to skull and brain injuries, had a higher trauma score, more frequently died at the crime scene and had a shorter survival time.

## Supporting information


Figure S1‐S19
