# Supplementary material for: Blunt force homicides in Denmark 1992–2016
Source: J Forensic Sci. 2022 Aug 18;67(6):2343–50. doi: 10.1111/1556-4029.15118 (PMC9804827; doi:10.1111/1556-4029.15118)
Supplement: Supplementary file 1 — Figure S1‐S19 [file JFO-67-2343-s001.pdf]

Journal of Forensic Sciences

Blunt force homicides in Denmark 1992-2016

Thomsen, A.H., et al.

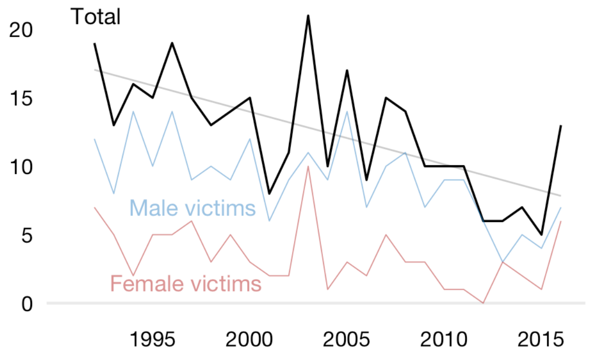

Supplementary figure 1: Blunt force homicides 1992-2016.

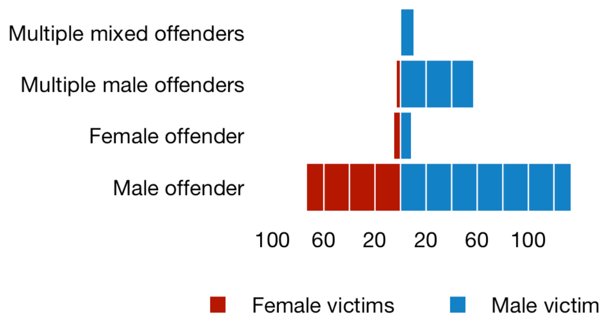

Supplementary figure 2: Offender sex related to sex of victim. The bars show the number of homicides for each main group.

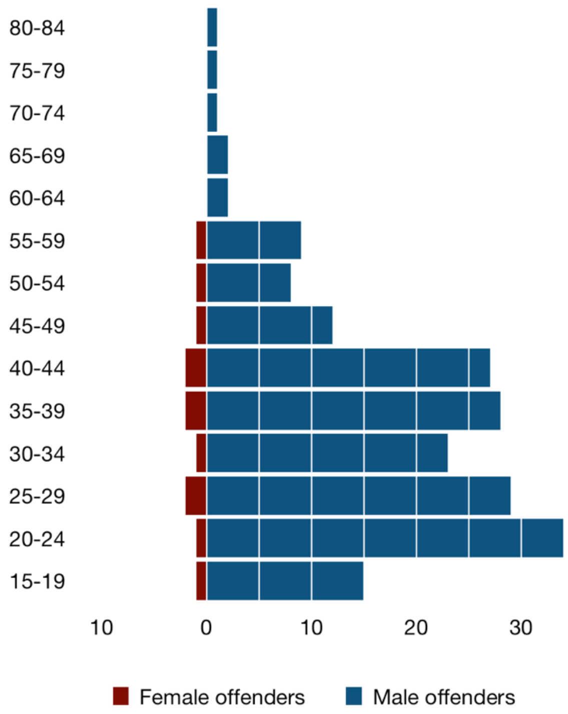

Supplementary figure 3: Age-sex pyramid for blunt force homicide offenders. The bars show the number of homicides for each age group in 5-year intervals.

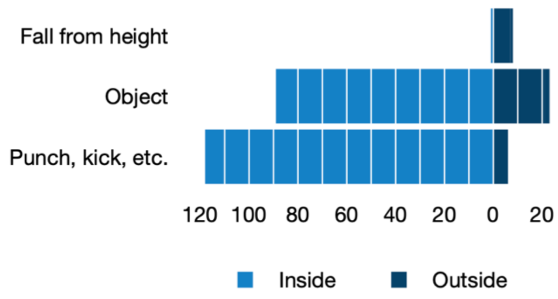

Supplementary figure 4: Blunt force homicide mechanism, grouped by location. The bars show the number of homicides for each main group.

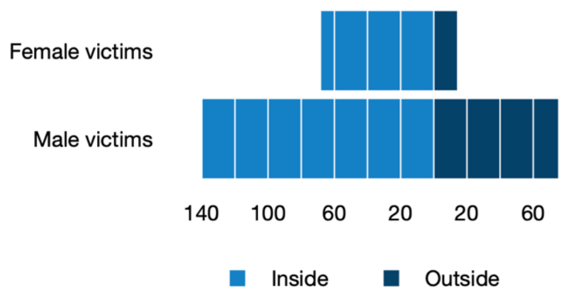

Supplementary figure 5: Victim sex, grouped by location. The bars show the number of homicides for each main group.

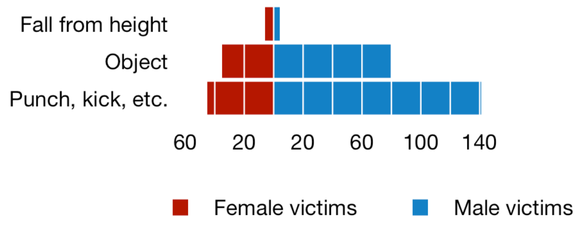

Supplementary figure 6: Blunt force homicide mechanism, grouped by victim sex. The bars show the number of homicides for each main group.

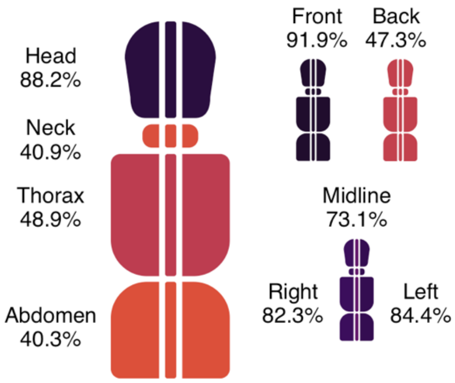

Supplementary figure 7: Distribution of contusions, abrasions, and lacerations relative to all 186 victims of **bodily force** homicide, i.e., the percentage of victims that have at least one lesion in a given area.

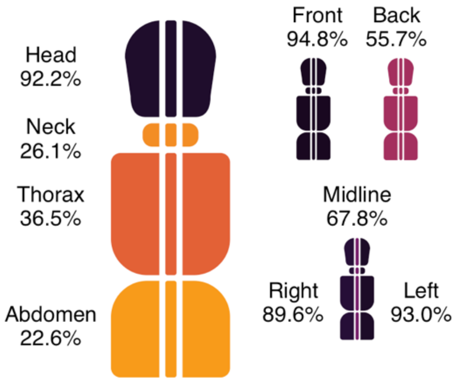

Supplementary figure 8: Distribution of contusions, abrasions, and lacerations relative to all 115 victims of **blunt object** homicide, i.e., the percentage of victims that have at least one lesion in a given area.

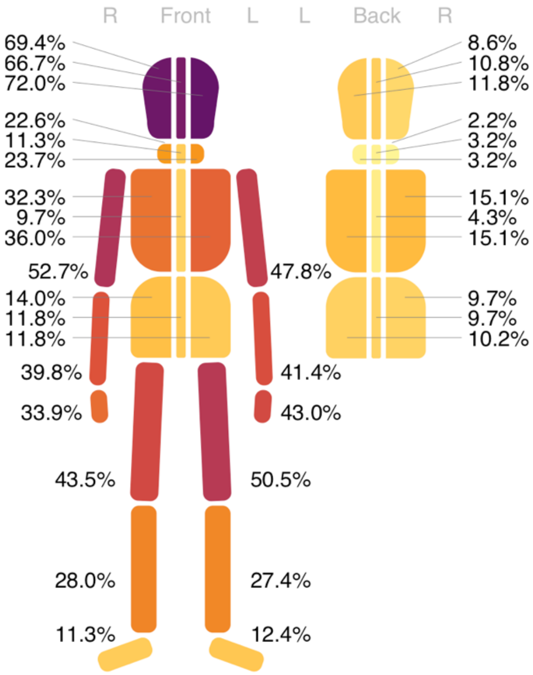

Supplementary figure 9: Distribution of contusions, abrasions, and lacerations relative to all 186 victims of **bodily force** homicide, i.e., the percentage of victims that have at least one lesion in a given area. Due to their mobility the extremities have not been separated into front and back.

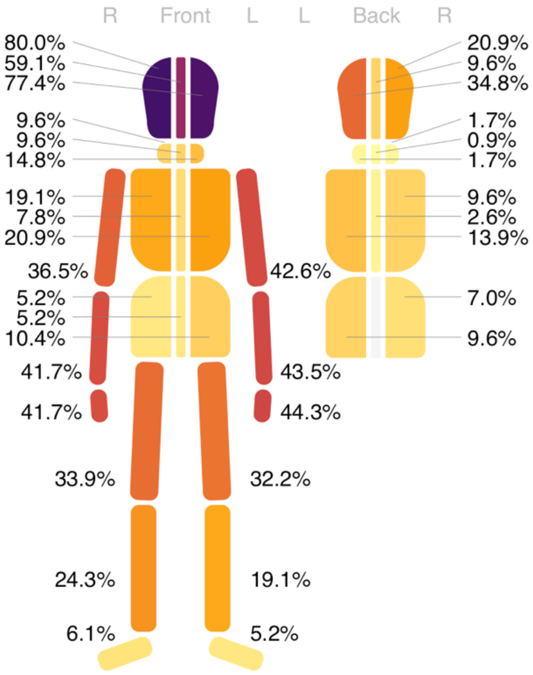

Supplementary figure 10: Distribution of contusions, abrasions, and lacerations relative to all 115 victims of **blunt object** homicide, i.e., the percentage of victims that have at least one lesion in a given area. Due to their mobility the extremities have not been separated into front and back.

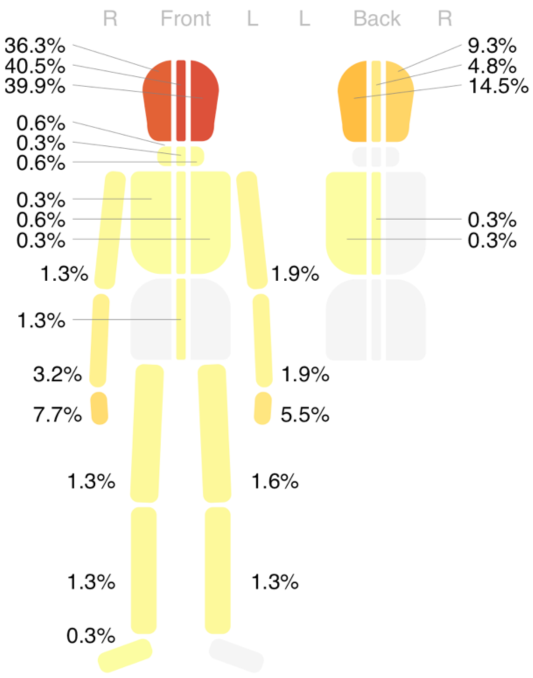

Supplementary figure 11: Distribution of **lacerations** relative to all 311 victims of blunt force homicide, i.e., the percentage of victims that have at least one lesion in a given area. Due to their mobility the extremities have not been separated into front and back.

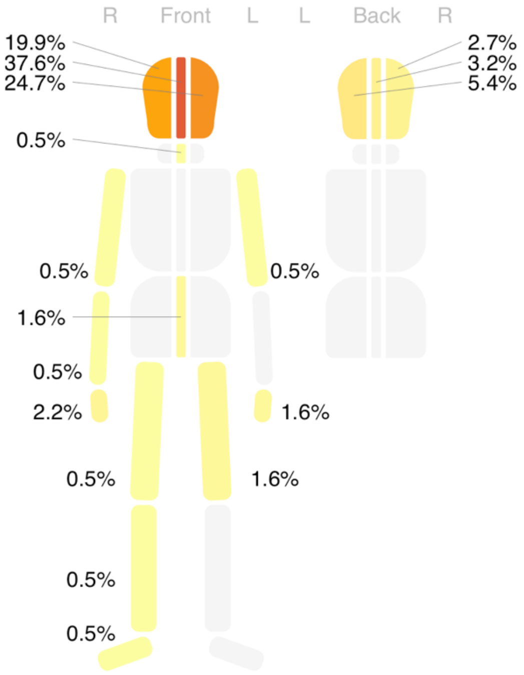

Supplementary figure 12: Distribution of **lacerations** relative to all 186 victims of **bodily force** homicide, i.e., the percentage of victims that have at least one lesion in a given area. Due to their mobility the extremities have not been separated into front and back.

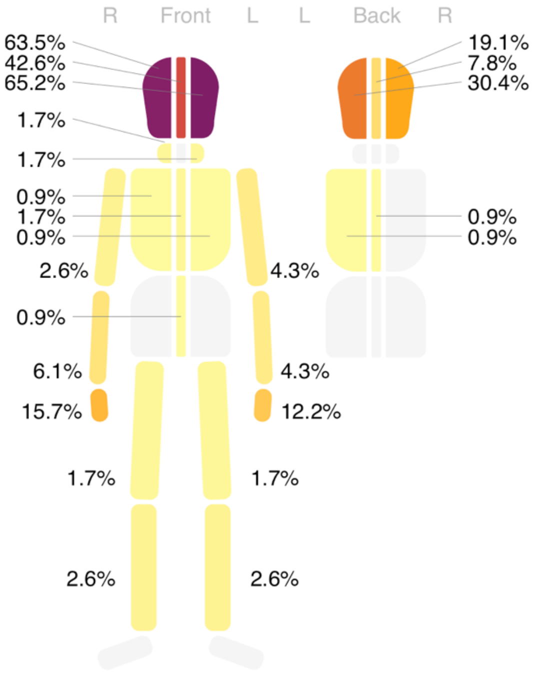

Supplementary figure 13: Distribution of **lacerations** relative to all 115 victims of **blunt object** homicide, i.e., the percentage of victims that have at least one lesion in a given area. Due to their mobility the extremities have not been separated into front and back.

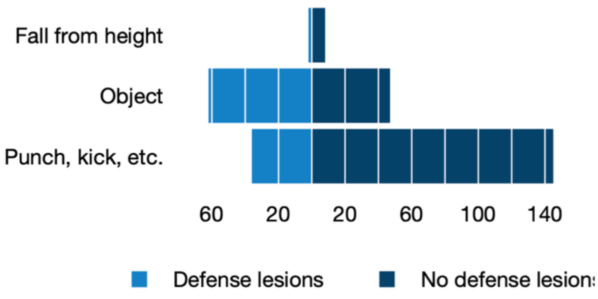

Supplementary figure 14: Blunt force homicide mechanism, grouped by findings of defense lesions. The bars show the number of homicides for each main group.

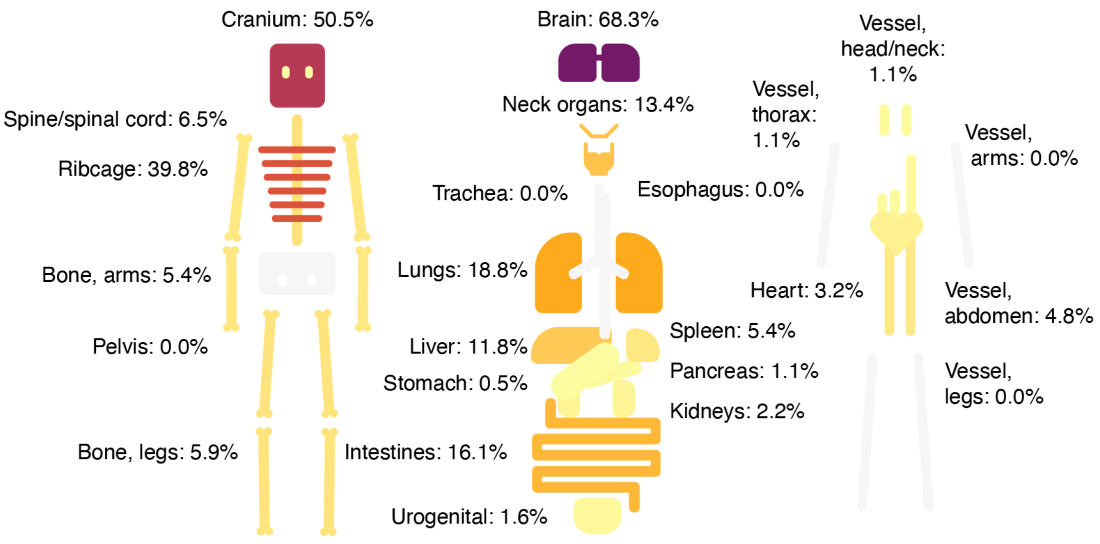

Supplementary figure 15: Distribution of injuries to organ systems, relative to all 186 victims of **bodily force** homicides, i.e., the percentage of victims that have at least one injury in a given organ.

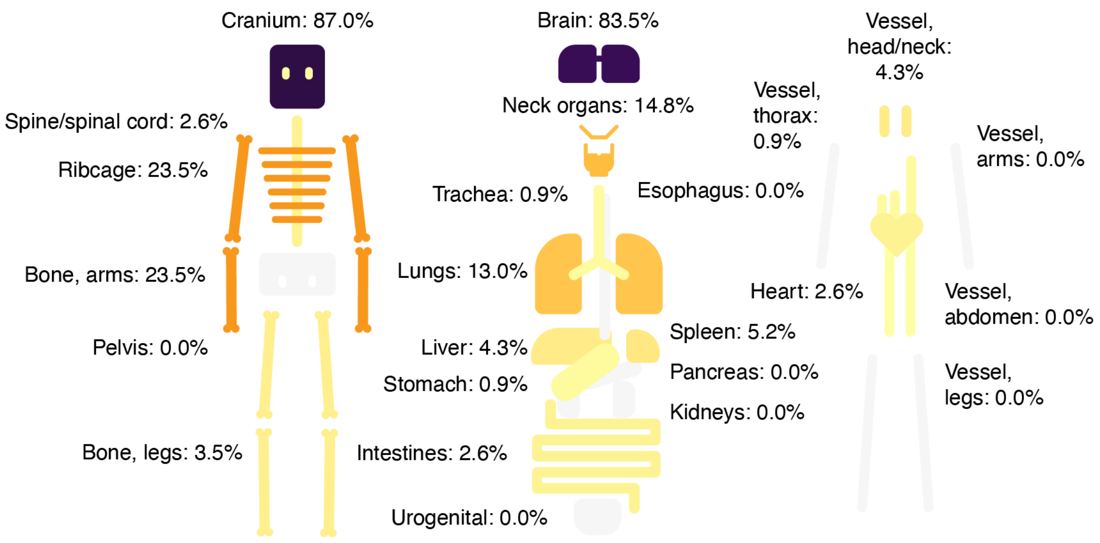

Supplementary figure 16: Distribution of injuries to organ systems, relative to all 115 victims of **blunt object** homicides, i.e., the percentage of victims that have at least one injury in a given organ.

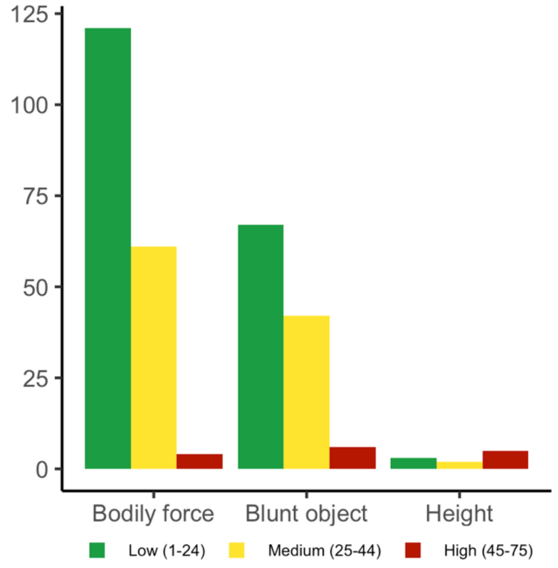

Supplementary figure 17: The number of victims ordered by ISS-group, grouped by victims killed with bodily force, blunt objects and fall from height.

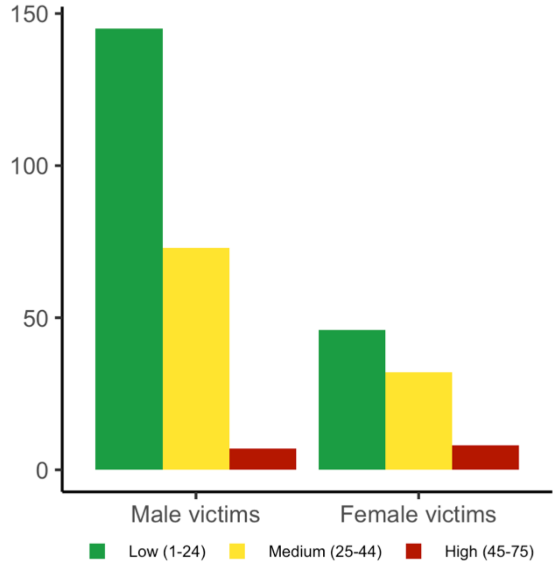

Supplementary figure 18: The number of victims ordered by ISS-group, grouped by victim sex.

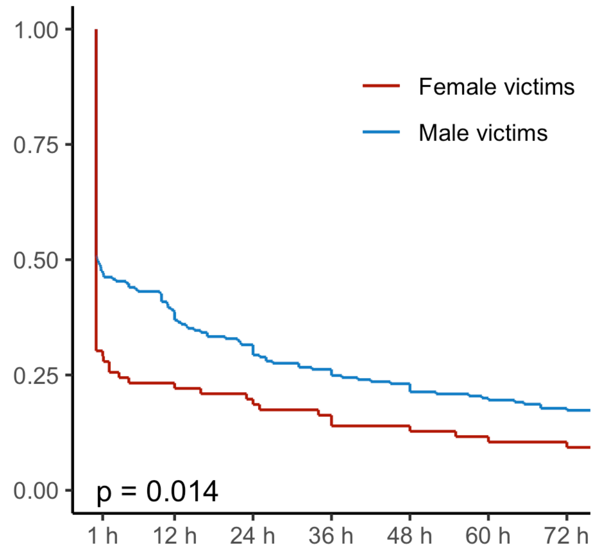

Supplementary figure 19: Survival curves for the first the 72 hours, grouped by victim sex.
